# SRFR1 Negatively Regulates Plant NB-LRR Resistance Protein Accumulation to Prevent Autoimmunity

**DOI:** 10.1371/journal.ppat.1001111

**Published:** 2010-09-16

**Authors:** Yingzhong Li, Shuxin Li, Dongling Bi, Yu Ti Cheng, Xin Li, Yuelin Zhang

**Affiliations:** 1 National Institute of Biological Sciences, Zhongguancun Life Science Park, Beijing, People's Republic of China; 2 College of Life Sciences, Peking University, Beijing, People's Republic of China; 3 Michael Smith Laboratories, University of British Columbia, Vancouver, British Columbia, Canada; RIKEN Plant Science, Japan

## Abstract

Plant defense responses need to be tightly regulated to prevent auto-immunity, which is detrimental to growth and development. To identify negative regulators of Resistance (R) protein-mediated resistance, we screened for mutants with constitutive defense responses in the *npr1-1* background. Map-based cloning revealed that one of the mutant genes encodes a conserved TPR domain-containing protein previously known as SRFR1 (SUPPRESSOR OF *rps4-RLD*). The constitutive defense responses in the *srfr1* mutants in Col-0 background are suppressed by mutations in *SNC1*, which encodes a TIR-NB-LRR (Toll Interleukin1 Receptor-Nucleotide Binding-Leu-Rich Repeat) R protein. Yeast two-hybrid screens identified SGT1a and SGT1b as interacting proteins of SRFR1. The interactions between SGT1 and SRFR1 were further confirmed by co-immunoprecipitation analysis. In *srfr1* mutants, levels of multiple NB-LRR R proteins including SNC1, RPS2 and RPS4 are increased. Increased accumulation of SNC1 is also observed in the *sgt1b* mutant. Our data suggest that SRFR1 functions together with SGT1 to negatively regulate R protein accumulation, which is required for preventing auto-activation of plant immunity.

## Author Summary

The nucleotide-binding domain and leucine-rich repeats-containing (NLR) proteins are structurally conserved immune receptors found in both animals and plants. Correct folding of NLR proteins requires two conserved proteins, SGT1 and HSP90. We showed that another evolutionarily conserved protein, SRFR1, interacts with SGT1 in both yeast two-hybrid assays and co-immunoprecipitation analysis. Loss-of-function mutations in *SRFR1* result in constitutive activation of immune responses. The constitutive activation of immune responses in the *srfr1* mutants is dependent on the NLR Resistance (R) protein SNC1. In *srfr1* mutant plants, levels of multiple R proteins including SNC1, RPS2 and RPS4 are elevated. Consistent with previous findings that SGT1b is involved in the negative regulation of protein levels of certain NLR R proteins, increased accumulation of SNC1 is also observed in the *sgt1b* mutant. Our data suggest that SRFR1 functions together with SGT1 to negatively regulate NLR R protein accumulation to prevent autoimmunity in plants.

## Introduction

To protect themselves from infections by microbial pathogens, plants have evolved a large number of immune receptors to sense pathogen-derived molecules and trigger defense responses [1]. Resistance (R) proteins with nucleotide-binding (NB) and Leucine-rich repeat (LRR) domains constitute the main type of intracellular plant immune receptors. In animals, similar nucleotide-binding domain and LRR-containing (NLR) proteins also function as intracellular immune receptors [2]. In plants, activation of NB-LRR R proteins often results in localized programmed cell death known as hypersensitive response (HR), accumulation of defense hormone salicylic acid (SA), and high expression of resistance marker genes termed *Pathogenesis-Related* (*PR*) genes [3].

Among the components that are required for R protein triggered immune responses, RAR1, HSP90 and SGT1 are three conserved proteins that function in correct folding and stabilization of NLR R proteins [4]. Loss of RAR1 function leads to compromised resistance mediated by multiple R proteins [5], [6], [7], [8], [9]. Accumulation of barley MLA proteins, potato Rx, and Arabidopsis RPM1 and RPS5 was reduced when RAR1 function was compromised [7], [10], [11]. Compromising the activity of HSP90 also caused reduced accumulation of several R proteins including RPM1, RPS5 and Rx [11], [12], [13]. The functions of SGT1 appear to be more complex. Silencing of *SGT1* in *Nicotiana benthamiana* resulted in reduced accumulation of Rx, suggesting that similar to RAR1 and HSP90, SGT1 is required for maintaining the protein level of Rx [14]. On the other hand, reduced accumulation of RPS5, but not RPM1 or RPS2, in the *rar1* mutant background can be suppressed by the *sgt1b* loss-of-function mutation. It was suggested that SGT1b antagonize RAR1 in regulating the accumulation of certain R proteins [11].

SGT1 contains three domains including the TPR (tetratricopeptide repeat) domain, the CS (present in CHP and SGT1 proteins) domain and the SGS (SGT1 specific) domain [4]. RAR1 contains two conserved cysteine and histidine rich domains named CHORD-I and CHORD-II [5]. Both SGT1 and RAR1 function as cochaperones of HSP90 [15], [16], [17]. The CS domain of SGT1 and CHORD-I domain of RAR1 bind to HSP90. The CHORDII domain of RAR1 binds to SGT1. In Arabidopsis genome, there are two copies of *SGT1* genes, *SGT1a* and *SGT1b*. Loss of the function of both genes lead to lethality [14]. Both plant and animal NLR proteins are substrates of the HSP90-RAR1-SGT1 chaperone complex [4]. Binding of SGT1 to these substrates is probably through the SGS domain in SGT1 and LRRs in NLRs [10].

Arabidopsis *SNC1* encodes a TIR-NB-LRR type of R protein [18]. In the *snc1* mutant, a gain-of-function mutation located in the region between NB and LRR constitutively activates downstream defense responses. *snc1* mutant plants exhibit dwarf morphology, accumulate high levels of salicylic acid (SA), and constitutively express *pathogenesis-related* (*PR*) genes and resistance to pathogens [19]. Overexpression of *SNC1* also results in constitutive activation of defense responses [20]. A recent report showed that the expression of *SNC1* is regulated at chromatin level by *MOS1*, which encodes a large protein with a conserved BAT2 domain [21].

Because autoimmunity is detrimental to plant growth and development, R protein mediated immunity is subjected to tight control. Since overexpression of *R* genes often leads to constitutive activation of defense responses [20], [22], transcription of *R* genes need to be controlled properly to keep R protein levels below a threshold to avoid constitutive activation of R protein-mediated immune responses. At protein level, without the presence of the microbial pathogens, R proteins are kept in an auto-inhibited conformation through intramolecular interactions [23]. Here we report that an SGT1-interacting protein negatively regulates R protein accumulation to prevent auto-activation of immune responses.

## Results

### Identification and characterization of the *snc5-1 npr1-1* mutant

**Figure 1:**
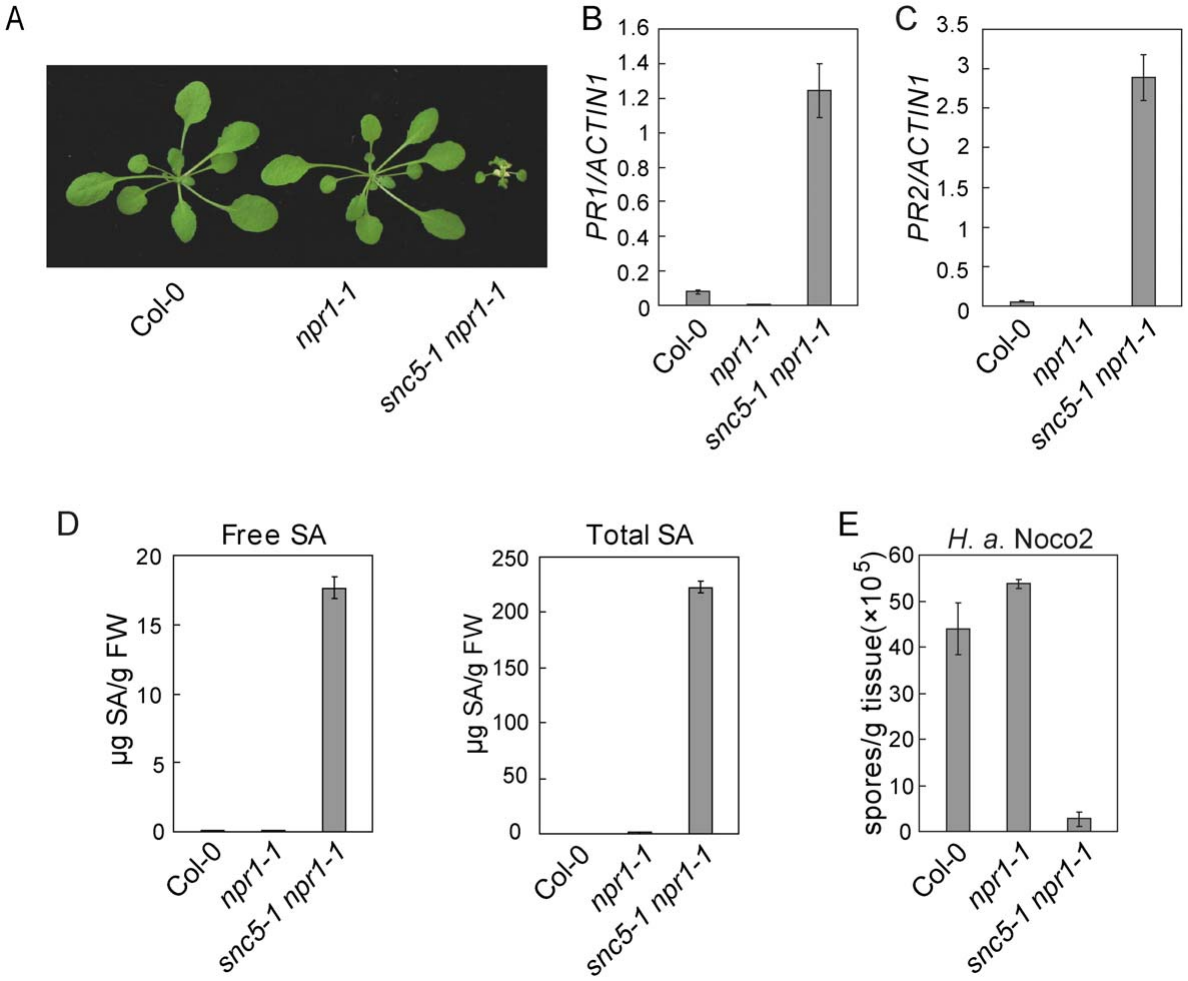
Defense responses are constitutively activated in *snc5-1 npr1-1*. (A) Morphology of wild type (Col-0), *npr1-1* and *snc5-1 npr1-1* plants grown on soil. The picture was taken when the plants were four weeks old. (B–C) Expression levels of *PR1* (B) and *PR2* (C) in wild type, *npr1-1* and *snc5-1 npr1-1* compared to *Actin 1*. Error bars represent standard deviation from three measurements. (D) Free and total SA levels in wild type (Col-0), *npr1-1* and *snc5-1 npr1-1*. Error bars represent standard deviation from four measurements. (E) Growth of *H. a.* Noco2 on wild type (Col-0), *npr1-1* and *snc5-1 npr1-1*. Error bars represent standard deviation from three measurements.

In Arabidopsis, NPR1 (*Nonexpresser of PR genes 1*) is an essential signaling component downstream of SA [24]. To search for negative regulators of defense responses independent of NPR1, an *npr1-1* suppressor screen was previously conducted [25]. A mutant named *snc5-1 npr1-1* was found to constitutively express the *BGL2 (PR2) Promoter-GUS* reporter gene in the *npr1-1* mutant background (Figure S1). *snc5-1 npr1-1* exhibited a dwarf morphology (Figure 1A) similar to *snc1*, an auto-activated TIR-NB-LRR *R* gene mutant identified in an independent *npr1-1* suppressor screen [19]. Plants heterozygous for *snc5-1 npr1-1* displayed wild type morphology, indicating that the *snc5-1* mutation is recessive.

In *snc5-1 npr1-1* mutant plants, both *PR1* and *PR2* were constitutively expressed (Figure 1B and 1C). To test whether *snc5-1 npr1-1* over-accumulates SA, SA levels in *snc5-1 npr1-1* and wild type plants were measured with high-performance liquid chromatography (HPLC). As shown in Figure 1D, both free and total SA (free SA plus glucose-conjugated SA) levels in *snc5-1 npr1-1* plants were much higher than in wild type controls.

Since the defense marker *PR* genes were activated in *snc5-1 npr1-1*, we tested whether *snc5-1 npr1-1* has enhanced pathogen resistance. *snc5-1 npr1-1* seedlings were challenged with *Hyaloperonospora arabidopsidis* Noco2 (*H. a.* Noco2), an oomycete downy mildew pathogen virulent on Arabidopsis Col-0 ecotype. As shown in Figure 1E, sporulation of *H. a.* Noco2 on *snc5-1 npr1-1* plants was much less than on wild type plants, indicating that defense responses are constitutively activated in *snc5-1 npr1-1*.

### Map-based cloning of *snc5-1*

To map the *snc5-1* mutation, *snc5-1 npr1-1* (in the Col-0 ecotype) was crossed with the wild type Ler ecotype to generate a segregating F2 population. In the F2 progeny, plants homozygous at the *snc5-1* locus were identified based on the dwarf morphology of *snc5-1*. Interestingly, the percentage of plants with dwarf morphology in the F2 population was less than one quarter, suggesting that there may be a natural modifier of *snc5-1* in Ler. Crude mapping using 24 dwarf plants suggested that two loci are required for the mutant phenotype: one is closely linked to the lower arm of chromosome 4 (marker F19F18 at 17.7 MB) and the other is linked to the middle of chromosome 4 (marker FCA5, at 9 MB).

**Figure 2:**
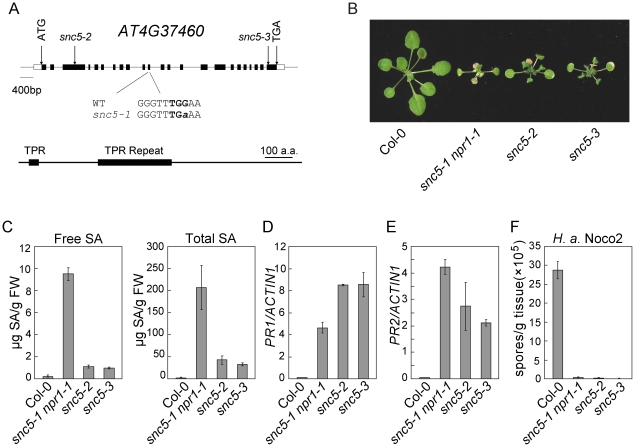
Map-based cloning of *snc5-1*. (A) Gene structure of *SNC5* (*At4g37460*) and positions of the molecular lesions in *snc5-1*, *snc5-2* (SAIL_412_E08) and *snc5-3* (SAIL_216_F11). Boxes are exons and lines indicate introns. (B) Morphology of wild type (Col-0), *snc5-1 npr1-1*, *snc5-2* and *snc5-3* plants grown on soil. The picture was taken when the plants were four weeks old. (C) Free and total SA levels in wild type (Col-0), *snc5-1 npr1-1*, *snc5-2* and *snc5-3*. Error bars represent standard deviation from four measurements. (D–E) Expression levels of *PR1* (E) and *PR2* (F) in wild type (Col-0), *snc5-1 npr1-1*, *snc5-2* and *snc5-3* compared to *Actin 1*. Error bars represent standard deviation from three measurements. (F) Growth of *H. a.* Noco2 spores on wild type (Col-0), *snc5-1 npr1-1*, *snc5-2* and *snc5-3*. Error bars represent standard deviation from three measurements.

For fine mapping of the locus on the lower arm of chromosome 4, we first identified F2 plants homozygous for the Col-0 sequence at marker FCA5 and heterozygous at marker F19F18. About 500 F3 plants from these F2 lines were genotyped with the markers T16L1 and F19F18. The *snc5-1* mutation was further mapped to a 92 kb region between markers F6G17 and F19F18 after analyzing the recombinants between T16L1 and F19F18. Sequence analysis of the genes in this region identified a single G to A mutation in *At4G37460*, which introduces an early stop codon in the middle of the protein (Figure 2A). *At4G37460* was predicted to encode a TPR domain-containing protein. Analysis of *At4G37460* expression using the microarray database at The Arabidopsis Information Resource found that it is expressed in all tissues.

To confirm the mutation in *At4G37460* causes the activation of defense responses, we analyzed two additional T-DNA knockout alleles of *At4G37460*, *snc5-2* (SAIL_412_E08) and *snc5-3* (SAIL_216_F11), both carrying T-DNA insertions in exons of *At4g37460* (Figure 2A). These two mutants showed similar dwarf morphology as *snc5-1 npr1-1* (Figure 2B). RT-PCR analysis showed that full length *At4G37460* was no longer expressed in the two T-DNA mutants (Figure S2). Both mutants accumulated high levels of SA (Figure 2C). Consistent with previous reports that NPR1 functions in negative feedback regulation of SA accumulation [19], [26], the *snc5-1 npr1-1* double mutant accumulated higher levels of SA than the *snc5-2* and *snc5-3* single mutants. Like *snc5-1 npr1-1*, *snc5-2* and *snc5-3* also constitutively expressed *PR1* (Figure 2D) and *PR2* (Figure 2E) and exhibited enhanced resistance to *H. a.* Noco2 (Figure 2F), suggesting that the mutations in *At4G37460* cause the activation of defense responses. It also indicates that the locus in the middle of chromosome 4 is probably a natural modifier of *snc5-1*.

Recently it was reported that mutants of *At4g37460* named *srfr1* (*suppressors of rps4-RLD*) in the RLD ecotype background exhibited enhanced resistance against *Pseudomonas syringae pv. tomato* DC3000 expressing *avrRps4* [27]. Unlike the *snc5* mutants in Col background, defense responses are not constitutively activated in the *srfr1* mutants identified in RLD ecotype and these mutants remain fully susceptible to the virulent *P.s.t.* DC3000 strain without *avrRps4* [28].

To be consistent with the literature, we renamed *snc5-1*, *snc5-2* and *snc5-3* in the Columbia background as *srfr1-3*, *srfr1-4* and *srfr1-5*, respectively. The protein encoded by *At4g37460* is referred to as SRFR1. Sequence analysis revealed that SRFR1 is conserved in plants and vertebrates (Figure S3), but not present in yeast and invertebrates such as *C. elegans* and *D. melanogaster*. The biochemical function of the protein is unknown.

### *snc5-1*/*srfr1-3* activates *SNC1*-mediated resistance

To further map the modifying locus affecting *srfr1-3 npr1-1* mutant morphology, we identified F2 plants that are homozygous for Col-0 at marker F19F18 (close to *SRFR1*) and heterozygous at marker FCA5 (close to the modifier). About 500 F3 plants from these lines were genotyped using the markers FCA5 and F1N20. The modifier was further mapped to the region between marker FCA6 and FCA8 after analyzing the recombinants between FCA5 and F1N20. This region contains the *RPP4 R*-gene cluster (Parker et al. 1997), which *SNC1* is a member of.

**Figure 3:**
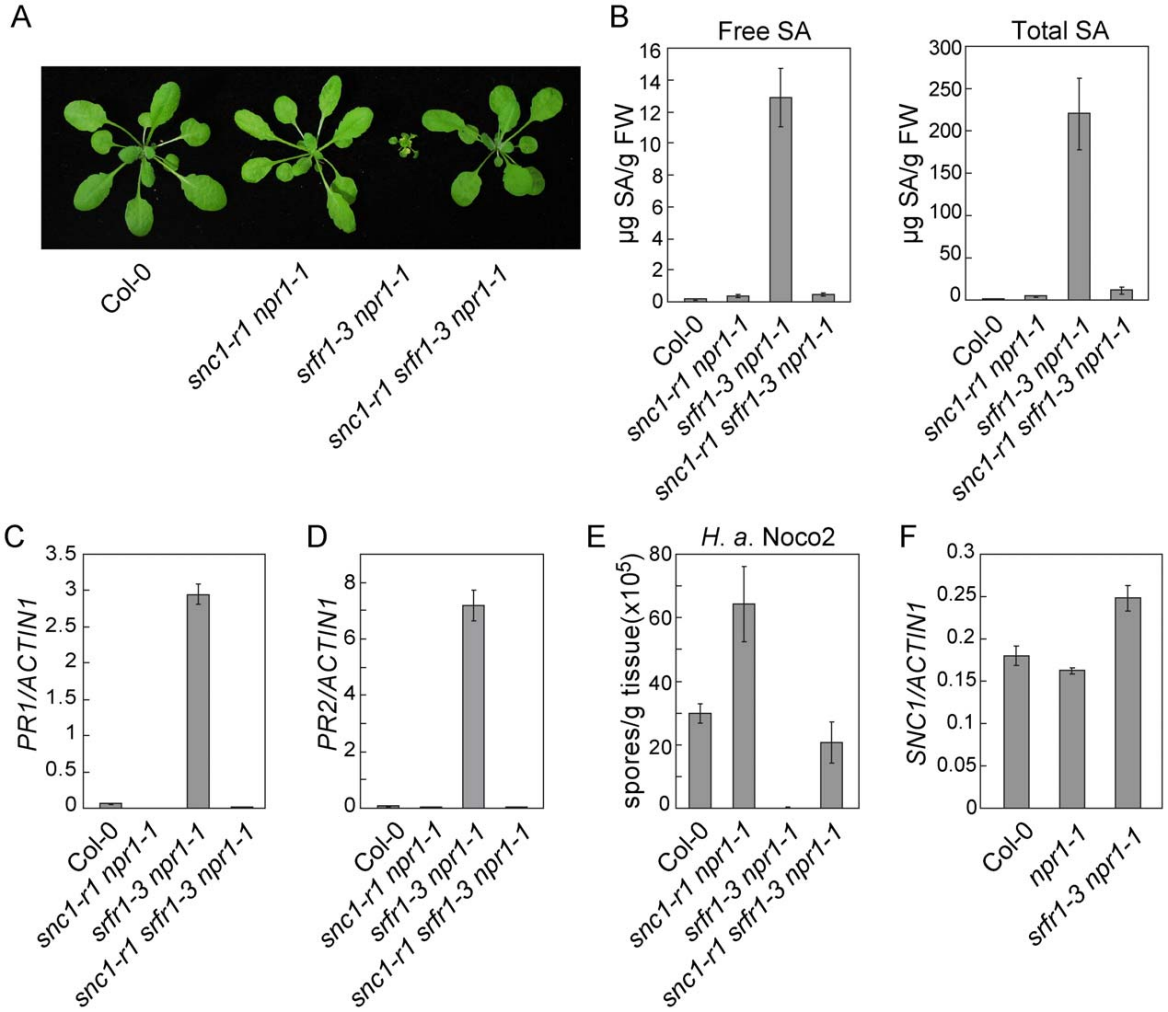
Loss of *SNC1* function suppresses constitutive defense responses in *snc5-1*/*srfr1-3*. (A) Morphology of wild type (Col-0), *snc1-r1 npr1-1*, *srfr1-3 npr1-1* and *snc1-r1 srfr1-3 npr1-1* plants grown on soil. The picture was taken when the plants were four weeks old. (B) Free and total SA in wild type (Col-0), *snc1-r1 npr1-1*, *srfr1-3 npr1-1* and *snc1-r1 srfr1-3 npr1-1*. Error bars represent standard deviation from four measurements. (C–D) Expression levels of *PR1* (C) and *PR2* (D) in wild type (Col-0), *snc1-r1 npr1-1*, *srfr1-3 npr1-1* and *snc1-r1 srfr1-3 npr1-1* compared to *Actin1*. Error bars represent standard deviation from three measurements. (E) Growth of *H. a.* Noco2 on wild type (Col-0), *snc1-r1 npr1-1*, *srfr1-3 npr1-1* and *snc1-r1 srfr1-3 npr1-1*. Error bars represent standard deviation from three measurements. (F) Expression levels of *SNC1* in wild type (Col-0), *npr1-1* and *srfr1-3 npr1-1* determined by q-RT-PCR. Error bars represent standard deviation from three measurements.

To identify the modifier required for the mutant phenotypes of *srfr1-3 npr1-1*, we mutagenized *srfr1-3 npr1-1* with EMS and looked for suppressors of *srfr1-3 npr1-1*. Because the *SNC1* locus is highly polymorphic in different ecotypes [29], we hypothesized that it may be the natural modifier. When we sequenced the *SNC1* locus in four of the suppressor mutants, we found that two of them contained mutations in *SNC1* (Figure S4). To confirm that *SNC1* is indeed the modifier of *srfr1-3 npr1-1*, we crossed *snc1-r1*, a known null mutant allele of *SNC1* containing a deletion of 8 bp in the first exon [18], into *srfr1-3 npr1-1*. We found that the *snc1-r1 srfr1-3 npr1-1* mutant plants displayed wild type morphology (Figure 3A), a stronger suppression compared to mutant alleles with point mutations identified from the *srfr1-3* suppressor screen. Further analysis of the triple mutant showed that the elevated SA levels (Figure 3B), constitutive expression of *PR* genes (Figure 3C–3D) and resistance to *H. a.* Noco2 (Figure 3E) in *srfr1-3 npr1-1* were also blocked by the *snc1-r1* mutation, suggesting that *srfr1-3* activates *SNC1*-mediated resistance pathways.

To test whether activation of defense responses in *srfr1-3 npr1-1* was caused by overexpression of *SNC1* at transcription level, the expression level of *SNC1* was determined by real-time RT-PCR. As shown in Figure 3F, *SNC1* expression in *srfr1-3 npr1-1* is only slightly higher than that in wild type and *npr1-1* plants. The small increase in *SNC1* transcript level probably is not the cause of the dramatic phenotypes observed in *srfr1-3 npr1-1*.

Interestingly, the *snc1-r1 srfr1-3 npr1-1* triple mutant is less susceptible to *H. a.* Noco2 than the *snc1-r1 npr1-1* double mutant (Figure 3E), suggesting that *srfr1-3* may also affect *SNC1*-independent resistance responses. To test whether *srfr1-3* affects resistance specified by additional *R* genes, we analyzed resistance mediated by *RPP4*, *RPS2* and *RPS4* in *snc1-r1 srfr1-3 npr1-1*. As shown in Figure S5A, the *snc1-r1 srfr1-3 npr1-1* triple mutant displayed enhanced resistance to *H. a.* Emwa1 comparing to the *snc1-r1 npr1-1* double mutant, suggesting that the *srfr1-3* mutation enhances *RPP4*-mediated resistance. In addition, *snc1-r1 srfr1-3 npr1-1* exhibited enhanced resistance to *P.s.t.* DC3000 carrying *avrRpt2* or *avrRps4* comparing to *npr1-1* (Figure S5B and S5C), indicating that resistance mediated by *RPS2* and *RPS4* is also enhanced by the *srfr1-3* mutation.

### SRFR1/SNC5 interacts with SGT1a and SGT1b

**Figure 4:**
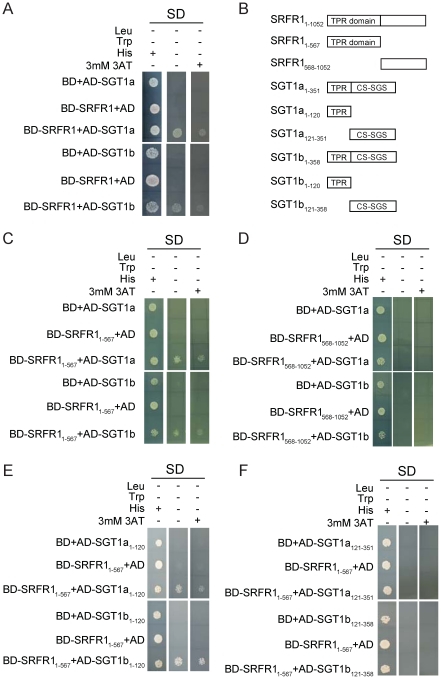
SRFR1 interacts with SGT1a and SGT1b in yeast two-hybrid analysis. (A) Interactions between full-length SRFR1 with SGT1a and SGT1b. (B) A diagram of the truncated proteins of SRFR1, SGT1a and SGT1b expressed in yeast two-hybrid vectors. (C–D) Interactions between the TPR domain (C) and C-terminal part (D) of SRFR1 with SGT1a and SGT1b. (E–F) Interactions between the TPR domain of SRFR1 with the TPR domains (E) or CS-SGS domains of SGT1a and SGT1b.

SRFR1 contains a TPR domain at its N-terminal half and a conserved C-terminal domain with unknown function. Since TPR domains are often involved in protein-protein interactions, SRFR1 probably functions through association with other proteins. To identify interacting partners with SRFR1, we performed a yeast two-hybrid screen using the full-length SRFR1 as bait. Seven positive cDNA clones were identified on synthetic dropout plates lacking Histidine (data not shown). Sequence analysis showed that one clone contained *SGT1a* (encoding amino acid 1-351) and another contained *SGT1b* (encoding amino acid 6-358) cDNA. To confirm the interactions between SRFR1 and SGT1a/b, the cDNA clones were recovered from yeast and used for additional assays. As shown in Figure 4A, both SGT1a and SGT1b interact with SRFR1 but not the empty vectors in the yeast two-hybrid assays. β-Gal assays were also performed to confirm the interactions (data not shown).

To determine which parts of SRFR1 and SGT1a/1b interact with each other, we created a series of deletion constructs of SRFR1 and SGT1a/1b (Figure 4B). As shown in Figure 4C and 4D, the N-terminal TPR domain but not the C-terminal half of SRFR1 interacted with SGT1a and SGT1b, suggesting that SRFR1 interacts with SGT1 through its TPR domain. When the TPR domain of SRFR1 was expressed together with the truncated SGT1a/1b proteins, it was found to interact with the TPR domains of SGT1a and SGT1b (Figure 4E), but not with the CS plus SGS domains in the yeast two-hybrid assay (Figure 4F). These interactions were further confirmed by β-Gal assays (data not shown). We also tested whether the TPR domain of SRFR1 self-associates in the yeast two-hybrid assays. As shown in Figure S6, the TPR domain of SRFR1 interacts with the TPR domain of SGT1b but not itself.

### SRFR1/SNC5 associates with SGT1 *in planta*

**Figure 5:**
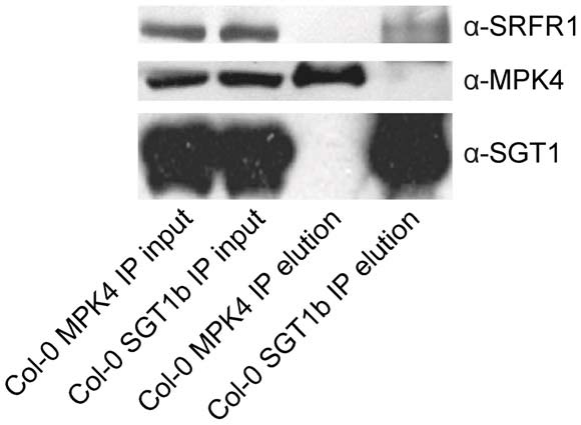
SGT1 associates with SRFR1 *in planta*. Co-IP of SGT1 with SRFR1 in total protein extracts from wild type Col-0 plants. Protein extracts were subjected to immunoprecipitation with anti-SGT1b or anti-MPK4 agarose beads. Crude lysates (left panels, Input) and immunoprecipitated proteins (right panels) were detected with Anti-SRFR1, anti-SGT1 or anti-MPK4 antibodies.

To test whether SRFR1 and SGT1 associate with each other *in planta*, we conducted co-immunoprecipitation (co-IP) analysis. First, we generated a polyclonal antibody against SRFR1, which has a predicted size of 118 kD. The anti-SRFR1 antiserum detected a protein around 120 kD present in wild type but not the *srfr1-3 npr1-1* or *srfr1-4* mutant plants (Figure S7), indicating that the antibody specifically detects SRFR1. Next we performed IP experiments using an anti-SGT1 antibody that can detect both SGT1a and SGT1b. As a control, we also performed IP using an anti-MPK4 antibody. Both SRFR1 and MPK4 were localized to cytosol and nucleus (Figure S8). Proteins that were immunoprecipitated by the antibodies were subsequently detected by western blot analysis using the SGT1, MPK4 or SRFR1 antibodies. As shown in Figure 5, SRFR1 co-immunoprecipitates with SGT1, but not with MPK4, indicating that SRFR1 and SGT1 associate with each other *in planta*.

### The SNC1 protein level is elevated in *snc5*/*srfr1* mutants

**Figure 6:**
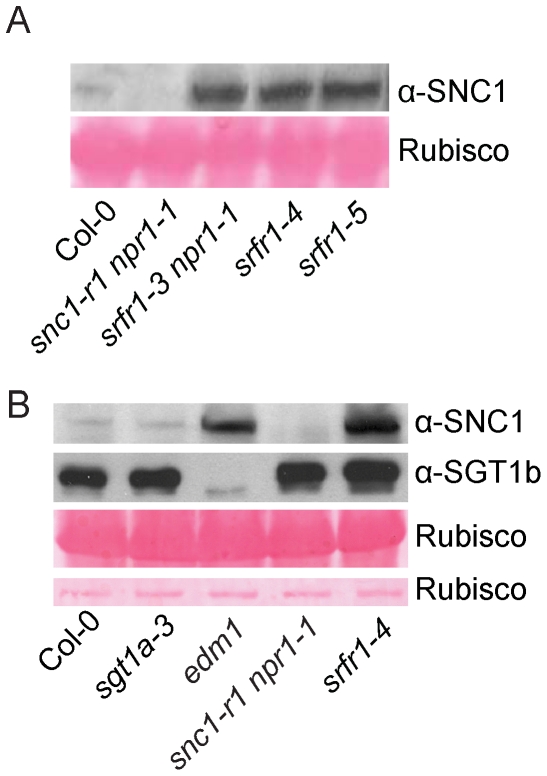
SNC1 protein levels in *srfr1* mutant plants. (A) Western blot analysis of SNC1 protein levels in total protein extracts of wild type (Col-0), *snc1-r1 npr1-1*, *srfr1-3 npr1-1*, *srfr1-4* and *srfr1-5*. (B) Western blot analysis of SNC1 protein levels in total protein extracts of wild type (Col-0), *sgt1a-3*, *edm1-1* (*sgt1b*), *snc1-r1 npr1-1* and *srfr1-4*.

Since SRFR1/SNC5 interacts with SGT1 and SGT1 has been shown to regulate R protein stability through its association with RAR1 and HSP90, we tested whether the accumulation of SNC1 is affected in the *srfr1* mutants. We generated a SNC1-specific antibody against a peptide unique in the SNC1 protein. SNC1 has a predicted size of 147 kD. The anti-SNC1 antibody detected a protein around 150 kD in the wild type, but not in the *snc1-r1* deletion mutant (Figure 6A), indicating that the antibody is specific against SNC1. In the *srfr1-3 npr1-1*, *srfr1-4* and *srfr1-5* mutant plants, SNC1 protein levels are much higher than that in the wild type plants, suggesting that loss of the function of SRFR1 results in over-accumulation of SNC1. To test whether mutations in *SGT1b* and *SGT1a* affect the accumulation of SNC1, we also analyzed the SNC1 protein levels in the *sgt1b* deletion allele *edm1-1* [30] and *sgt1a-3*, a T-DNA knockout allele of *sgt1a*. Real-time RT-PCR showed that the expression of *SGT1a* was dramatically decreased in *sgt1a-3* (Figure S9). We observed increased accumulation of SNC1 protein in *edm1-1*, but not in *sgt1a-3* (Figure 6B). Taken together, both SRFR1 and SGT1b contribute to the negative regulation of SNC1 stability.

### RPS2 and RPS4 protein levels are elevated in *snc1-r1 srfr1-3* and *snc1-r1 srfr1-3 npr1-1*

**Figure 7:**
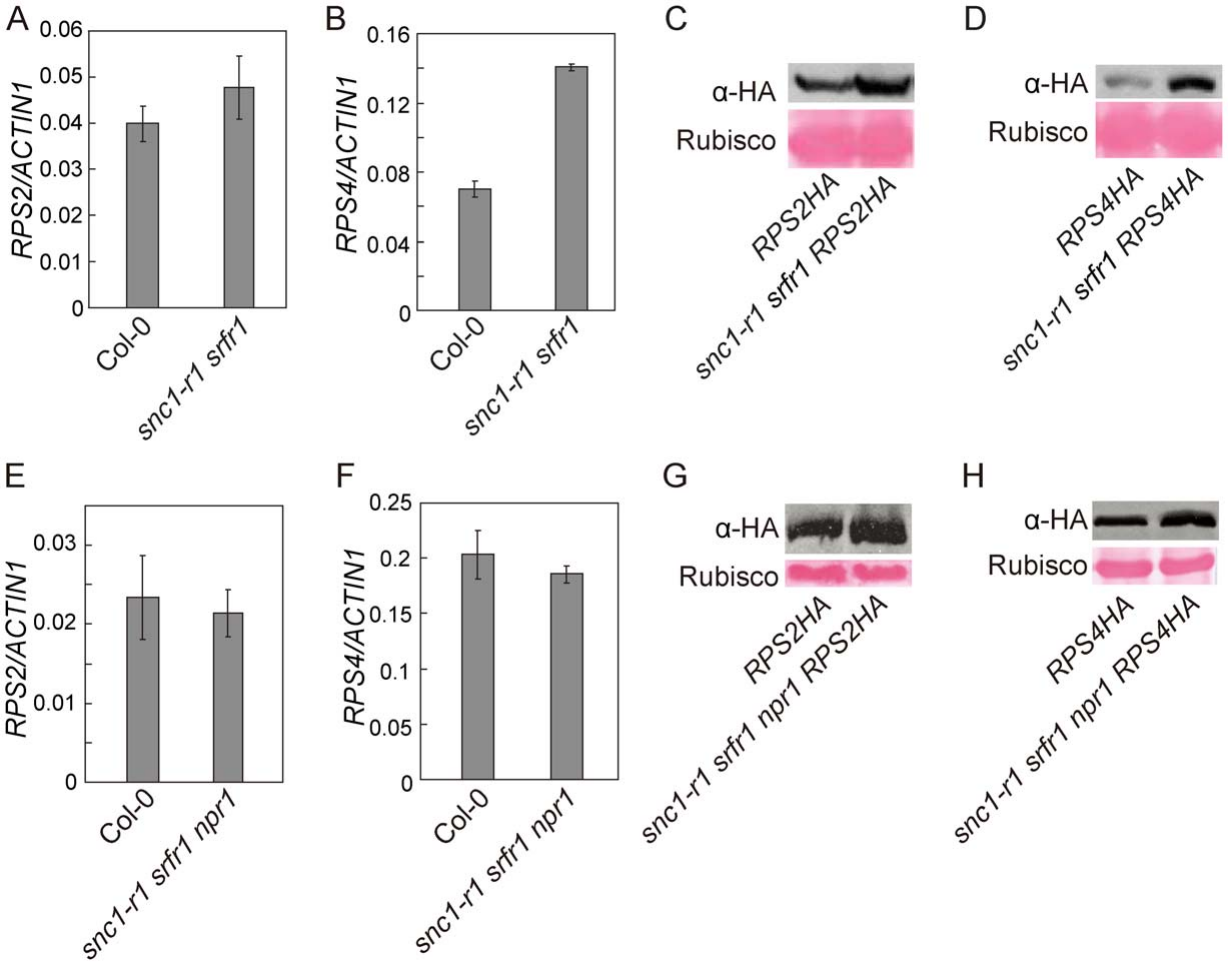
Analysis of the transcript levels of *RPS2* and *RPS4* and the accumulation of RPS2-HA and RPS4-HA proteins in *snc1-r1 srfr1-3* and *snc1-r1 srfr1-3 npr1-1*. (A–B) Real-time RT-PCR analysis of *RPS2* (A) and *RPS4* (B) expression in wild type (Col-0) and *snc1-r1 srfr1-3*. Error bars represent standard deviation from three measurements. (C) Western blot analysis of RPS2-HA protein levels in total protein extracts of *RPS2-HA* and *snc1-r1 srfr1-3 RPS2-HA* lines. (D) Western blot analysis of RPS4-HA protein levels in total protein extracts of *RPS4-HA* and *snc1-r1 srfr1-3 RPS4-HA* lines. (E–F) Real-time RT-PCR analysis of *RPS2* (E) and *RPS4* (F) expression in wild type (Col-0) and *snc1-r1 srfr1-3 npr1-1*. Error bars represent standard deviation from three measurements. (G) Western blot analysis of RPS2-HA protein levels in total protein extracts of *RPS2-HA* and *snc1-r1 srfr1-3 npr1-1 RPS2-HA* lines. (H) Western blot analysis of RPS4-HA protein levels in total protein extracts of *RPS4-HA* and *snc1-r1 srfr1-3 npr1-1RPS4-HA* lines.

To test whether *srfr1* mutations affect the accumulation of RPS2 and RPS4 proteins, we crossed *RPS2-HA* or *RPS4-HA* transgenic lines, expressed under their native promoters [31], [32], into *snc1-r1 srfr1-3* and *snc1-r1 srfr1-3 npr1-1* backgrounds. The *snc1-r1* mutation was included in the analysis to avoid the effect of constitutive activation of defense responses on the accumulation of the R proteins. In the *snc1-r1 srfr1-3* plants, the transcript level of *RPS2* was similar to that in wild type plants whereas the transcript of *RPS4* was about twice as much as that in wild type plants (Figure 7A and 7B). As shown in Figure 7C and 7D, both RPS2-HA and RPS4-HA accumulated to higher levels in *snc1-r1 srfr1-3* than in wild type plants.

In the *snc1-r1 srfr1-3 npr1-1* triple mutant, the transcript levels of *RPS2* and *RPS4* were similar to those in wild type plants (Figure 7E and 7F). As shown in Figure 7G, RPS2-HA accumulated to a higher level in *snc1-r1 srfr1-3 npr1-1* than in wild type. Accumulation of RPS4-HA was also increased in the triple mutant (Figure 7H), but the increase was not as dramatic as that observed in the *snc1-r1 srfr1-3* double mutant, suggesting that the increased RPS4-HA protein level in *snc1-r1 srfr1-3* was partly due to increased transcription of *RPS4*. These data suggest that SRFR1 contributes to the regulation of RPS2 and RPS4 protein levels.

## Discussion

In a suppressor screen of *npr1-1* to search for negative regulators of immune responses, we identified *snc5-1*/*srfr1-3* that constitutively expresses *PR* genes and pathogen resistance. Since loss-of-function mutations in *SNC1* block activation of defense responses in *srfr1-3 npr1-1*, the resistance activated by *srfr1-3* is mediated by the R protein SNC1. In addition, SNC1 protein over-accumulated in *srfr1* mutants, suggesting that SRFR1 regulates the stability of SNC1 and over-accumulation of SNC1 caused the activation of immune responses. A previous study showed that *srfr1* mutants in the RLD ecotype background do not activate constitutive defense responses [28]. The lack of constitutive defense responses in the *srfr1* mutants is probably due to the absence of a functional *SNC1* gene in the RLD background, whereas the enhanced resistance to DC3000 with *avrRps4* may be caused by increased accumulation of an unidentified R protein that recognizes AvrRPS4.

From a yeast two-hybrid screen, we found that SRFR1 interacts with SGT1a and SGT1b. *In planta* interactions between SGT1 and SRFR1 were confirmed by co-IP experiments. Like in *srfr1* mutants, elevated SNC1 protein level was also observed in *edm1-1*, the deletion mutant allele of *sgt1b*. This is consistent with SRFR1 and SGT1 function together to regulate the stability of SNC1. Interestingly, the over-accumulation of SNC1 in *sgt1b* mutant plants does not cause constitutive activation of defense responses, suggesting that SNC1 protein over-accumulated in *sgt1b* mutant may have reduced activity. Since SGT1 may have dual functions in negative regulation of R protein accumulation as well as positive regulation of R protein folding [11], it is likely that the over-accumulated R proteins in *sgt1b* mutant are not folded correctly without the assistance of SGT1b, thus not able to trigger immune responses.

SGT1 contains three domains, the N-terminal TPR domain, the central CS and C-terminal SGS domain. The CS domain interacts with both RAR1 and HSP90 while the SGS domain may form contacts with the LRRs of R proteins [10], [16]. The function of the TPR domain is unclear. Interestingly, the TPR domain of SGT1 is missing in some non-plant species such as *C. elegans* [4], suggesting that the TPR domain may have a specialized function. Our study showed that the TPR domain of SGT1 interacts with SRFR1, suggesting that this domain may function in negative regulation of R protein accumulation, which is consistent with the association of SGT1 with components of the SCF (SKP1, Cullin, F-box protein) ubiquitin ligase complex [33], [34] and SGT1 is required for SCF-mediated auxin responses [34]. The TPR domain of SGT1b has previously been shown to be dispensable for the function of SGT1b in regulating R protein mediated resistance as well as auxin signaling when it was overexpressed [14]. It remains to be determined whether a truncated SGT1b without the TPR domain under its own promoter is able to complement the phenotypes of *sgt1b* as well as the embryo lethality phenotype in the *sgt1a sgt1b* double mutant.

Analysis of SGT1 functions in Arabidopsis has been complicated by the presence of two closely related SGT1 proteins with overlapping functions [14], [35]. STG1a is expressed at a lower level than SGT1b, but it has intrinsic activity to complement the *sgt1b* mutant when its expression is increased to a certain level. Thus the mutant phenotypes of *sgt1b* are probably results of partial loss of SGT1 functions. While SGT1b has been shown to be required for the function of a number of R proteins (reviewed by Shirasu [4]), RPS5 function is not affected in *sgt1b*. The SGT1a activity may be sufficient for proper folding of RPS5. An unexpected result is that a loss-of-function mutation in *SGT1b* suppresses the reduced accumulation of RPS5 and loss of RPS5 function in *rar1*, implicating that SGT1b may also play a role in the negative regulation of R protein accumulation [11]. Our data support the model proposed by Azevedo *et al*. [14] that SGT1 has dual functions in regulating R protein-mediated immune responses. In addition to its function as a co-chaperone of HSP90 in positively regulating R protein folding, it may also be involved in the negative regulation of R protein stability by association with SRFR1 through its N-terminal TPR domain.

In addition to SNC1, SRFR1 may regulate the accumulation of other R proteins. In the *srfr1-3* mutant plants, both RPS2-HA and RPS4-HA fusion proteins accumulate to higher levels than in wild type plants. Because knockout of *SNC1* is sufficient to block the constitutive defense responses in the *srfr1-3* mutant, the increased accumulation of other R proteins such as RPS2 and PRS4 probably has not reached the threshold levels that would cause activation of these R proteins. Since SRFR1 and SGT1 are both conserved in plants and animals and SGT1 is required for the functions of animal NLR proteins such as NOD1, NOD2 and NLRP3 [36], [37], it will be interesting to test whether the homologs of SRFR1 in animals also function as negative regulators of NLR protein-mediated immune responses.

## Materials and Methods

### Plant material and growth conditions

All plants were grown under 16 hour light at 23°C and 8 hour dark at 20°C. *srfr1-3 npr1-1* was identified from an EMS-mutagenized mutant population in the *npr1-1* mutant background as previously described [25]. *snc5-2/srfr1-4* (SAIL_412_E08), *snc5-3/srfr1-5* (SAIL_216_F11) and *sgt1a-3* (SALK_122139C) were obtained from the Arabidopsis Biological Resource Center (ABRC). Homozygous plants for *snc5-2/srfr1-4* were identified by PCR using primers 5′-tcatcactaattccgcaacg-3′ and 5′-cgacttatgtaacggatcag-3′. Homozygous plants for *snc5-3/srfr1-5* were identified by PCR using primers 5′-ctatggttctactgagctcg-3′ and 5′-tgctcatggtttagttagcc-3′. The *RPS2-HA* and *RPS4-HA* transgenic lines were described previously [31], [32].

*snc1-r1 npr1-1* is a deletion mutant of *snc1* described previously [18]. The *snc1-r1 srfr1-3 npr1-1* triple mutant was generated by crossing *snc1-r1 npr1-1* with *srfr1-3 npr1-1* and genotyping the F2 population. *srfr1-3* mutation were identified by PCR using primers 5′-caggggaagtaatcttatcggatatcac-3′ and 5′-caattttcctgtcttgaccagggttcg-3′ followed by digestion with *Taq*I. Plants homozygous for *snc1-r1* were identified by PCR using primers 10C-WT-F (5′-cctggtgcctgaatgaattg-3′) and 10C-R (5′-atcatccgatggtgtcatag-3′).

### Mutant characterization

Infection of *H. a.* Noco2 was carried out on two-week-old seedlings by spraying with spore suspensions at a concentration of 50,000 spores per ml of water. The plants were kept at 18°C in 12 h light/12 h dark cycles with 95% humidity. Infections were scored seven days post inoculation by counting the number of spores with a hemocytometer.

RNA was extracted from the 12-day-old seedlings grown on MS plates using the RNAiso reagent (Takara). Reverse transcription (RT) was performed using the M-MLV reverse transcriptase from Takara. For gene expression analysis, real-time PCR was carried out using the Perfect Real Time kit (Takara). The sequences of primers used for amplification of *PR-1*, *PR-2* and *Actin1* were described previously [38]. SA was extracted as previously described and measured using HPLC [39].

### Map-based cloning of *snc5-1*/*srfr1-3*

Markers used for mapping were designed based on the Monsanto Arabidopsis polymorphisms and Landsberg sequence collections [40]. The primer sequences for AP20 are 5′-gtcattttctaaaatccaatatgaccg-3′ and 5′-gacgacatattgcacattttcatattg-3′. Primers for F6G17 are 5′-cacttccctggtgcgtccaa-3′ and 5′-ggacagaagatacaggtgag-3′. The primer sequences for F19F18 are 5′-aatcaatgattctatatacacatg-3′ and 5′-gacgaagattgcttggtgag-3′. The primer sequences for FCA5 are 5′-aatgcggtgttacccatggc-3′ and 5′-actcttccgataaacttcctc-3′. The primer sequences for FCA8 are 5′-gtcttcctctgccatttcac-3′ and 5′-gttgcgaaaagcagagattg-3′. All the markers are based on Indel polymorphisms.

### Co-immunoprecipitation and antibodies

About 0.9 g of 12-day-old seedlings were ground in liquid nitrogen to fine powder and 0.9 ml of grinding buffer with 50 mM Tris-HCl (pH 7.5), 10 mM MgCl_2_, 150 mM NaCl, 0.1% NP40, 1 mM PMSF, and 1 x Protease Inhibitor Coctail (Roche, 11873580001) was added to the powder. The sample was resuspended, transferred to 1.5 ml tubes and spun at 21,000 g for 10 min at 4°C. The supernatant was transferred to a tube containing 20 µl Protein A agarose beads (GE Healthcare, 17-1279-03) for pre-cleaning. After rotating for 25 minutes, the sample was spun at 21,000 g for 5 min at 4°C. 40 µl of the supernatant was saved as input. Antibody was added to the rest of the supernatant and the sample was kept at 4°C with continuous rotation for 2–3 hours. 20 µl of Protein A agrose beads was subsequently added to the sample and kept at 4°C with continuous rotation for 1 hour. The beads were spun down at 4,000 rpm for 30 sec at 4°C. The beads were washed with 1 ml of grinding buffer for three times before immunoprecipitated proteins were eluted with 40 µl 2 x SDS loading buffer.

SGT1b and the TPR domain of SRFR1 (a. a. 1-567) were expressed in *E. coli* and used to generate the anti-SGT1b and anti-SRFR1 antibodies in rabbit. The Anti-SNC1 antibody was generated against an SNC1-specific peptide (KAKSEDEKQS). The anti-MPK4 antibody was from Sigma (A6979). The anti-HA antibody was from Roche (REF#11867423001). Nuclei-depleted (ΔN) and nuclear (N) protein extracts of wild type plants were prepared as previously described [41].

### Yeast two-hybrid screen

To create the SRFR1 bait plasmid, *SRFR1* cDNA was amplified by primers 5′-aaaactgcagggcccatgaggcctcaatcgttgtaagtgctaag-3′ and 5′-cgcggatccggccgtcaaggccaatggcgacggcgacggcgaca-3′ and cloned into pGBKT7 (Clontech). The plasmid was sequenced and transformed into the yeast strain Y1348. The Arabidopsis prey library in pGADT7 was kindly provided by Dr. Qi Xie. 40 µg of the library DNA were transformed to yeast strain containing the bait plasmid. The transformed yeast cells were plated on the SD-Leu-Trp-His containing 3 mM 3AT. DNA inserts from the positive clones were amplified by PCR using primers T7 and AD-seq-R (5′-agatggtgcacgatgcacag-3′). The DNA fragments from PCR were digested with *Hinf*I to group the positive clones into different classes. DNAs from representative clones were sequenced. The plasmids from selected positive clones were extracted and transformed into *E. coli* to amplify the DNA for further analysis. For yeast growth assays, overnight yeast cultures were diluted to different concentration and plated on SD-Leu-Trp and SD-Leu-Trp-His dropout plates.

To make the bait plasmid containing the N-terminal half of SRFR1 (a.a 1-567), the cDNA fragment was amplified using 5′-cgcggatccggccgtcaaggccaatggcgacggcgacggcgaca-3′ and 5′-aaaactgcaggcccatgaggcctcatgcatcaagttccacgtcaa-3′. To make the bait plasmid containing the C-terminal half of SRFR1 (a.a. 568-1052), the cDNA fragment was amplified using 5′-gcgggtacccatatggggccgtcaaggccagtggagaaatttgttcttc-3′ and 5′-aaaactgcagggcccatgaggcctcaatcgttgtaagtgctaag-3′. The DNA fragments were cloned into the pGBKT7.

*SGT1* fragments were amplified by PCR and cloned into the prey vector pGADT7. SGT1a-TPR_1-120_ was amplified using 5′-ccggaattcatggcgaaggagcttgctga-3′ and 5′-gccgaattctcgagtcattctgtgattagaaaattgc-3′. SGT1b_1-120_ was amplified using 5′-ccggaattcatggccaaggaattagcaga-3′ and 5′-gccgaattctcgagtcattcttctgcaatacgaagat-3′. SGT1a_121-351_ was amplified using 5′-ccggaattcgaagagaaagatttggttca-3′ and 5′-cgcggatcctcagatctcccatttcttga-3′. SGT1b_121-358_ was amplified using 5′-ccggaattcgagaaagatttggttcagcc-3′ and 5′-cgcggatcctcaatactcccacttcttga-3′. The bait and prey vectors expressing the SRFR1 and SGT1 fragments were co-transformed into the Y1348 strain for yeast two-hybrid assays.

## Supporting Information

Figure S1 GUS staining of *npr1-1* and *snc5-1 npr1-1*. Two-week-old seedlings grown on MS media were stained for GUS activity. Both *npr1-1* and *snc5-1 npr1-1* contain the *BGL2 (PR2) Promoter-GUS* reporter gene.
(0.22 MB PDF)

Figure S2 Location of the T-DNA insertions (A) and semi-quantitative RT-PCR analysis of *SNC5* expression in the T-DNA knockout mutants *snc5-2* and *snc5-3* (B). Primers F1 and R1 were used in PCR amplification of *snc5-2*. Primers F2 and R2 were used in PCR amplification of *snc5-3*. The locations of the primers are indicated in (A).
(0.26 MB PDF)

Figure S3 Alignment of SRFR1 and its homologs in rice, human and mouse. AtSRFR1, OsSRFR1, MmSRFR1, and HsSRFR1 represent NP_195462, NP_001058749, NP_663582, and NP_078801 respectively. The sequences were retrieved from NCBI and aligned by the ClustalX2. The aligned data were further analyzed by the BOXSHADE 3.21 (http://www.ch.embnet.org/software/BOX_form.html).
(0.84 MB PDF)

Figure S4 Two suppressor mutants of *srfr1-3 npr1-1* carrying mutations in *SNC1*. (A) Morphology of *snc1-12 srfr1-3 npr1-1* and *snc1-13 srfr1-3 npr1-1*. (B) Molecular lesions in *SNC1* identified from *snc1-12* and *snc1-13*.
(0.40 MB PDF)

Figure S5 Immunity mediated by *RPP4*, *RPS2* and *RPS4* is enhanced in *snc1-r1 srfr1-3 npr1-1*. (A) Growth of *H. a.* Emwa1 on WT (Col-0), *snc1-r1 npr1-1*, *snc1-r1 srfr1-3 npr1-1*, and *eds1-2* (Col). Two-week-old seedlings were sprayed with *H. a.* Emwa1 at a concentration of 50,000 spores per ml water. Infection was scored 7 days after inoculation by counting the number of spores per gram of tissue. Error bars represent standard deviations from three measurements. (B-C) Growth of *P.s.t.* DC3000 *avrRpt2* (B) and *P.s.t.* DC3000 *avrRps4* (C) on WT (Col-0), *snc1-r1*, *npr1-1* and *snc1-r1 srfr1-3 npr1-1*. Leaves of five-week old plants were infiltrated with *P.s.t.* DC3000 carrying *avrRpt2* or *avrRps4* (OD_600_ = 0.001). Bacterial growth was determined at Day 0 and Day 3. The values presented are averages of six replicates ± standard deviations (SD). *, *P*<0.001, significant difference from *npr1-1*.
(0.25 MB PDF)

Figure S6 Yeast two-hybrid analysis of self-association of the TPR domain of SRFR1.
(0.28 MB PDF)

Figure S7 Western blot analysis of the SRFR1 protein in wild type and *snc5* mutants using the anti-SRFR1 antibody.
(0.17 MB PDF)

Figure S8 Localization of SRFR1 and MPK4. Immunoblot analysis of SRFR1 and MPK4 in nuclei-depleted (ΔN) and nuclear (N) protein extracts of wild type plants. Equal proportions of nuclei-depleted and nuclear protein extracts were loaded. Anti-PEPC was used as a cytosolic marker, and anti-Histone H3 was used as a nuclear marker.
(0.47 MB PDF)

Figure S9 Location of the T-DNA insertion in *sgt1a-3* (A) and real-time RT-PCR analysis of *SGT1a* expression in wild type (Col-0) and *sgt1a-3* (B). Primers used for the PCR analysis are indicated in (A). Error bars represent standard deviation from three measurements.
(0.23 MB PDF)
