# Supplementary figures and images for: SRFR1 Negatively Regulates Plant NB-LRR Resistance Protein Accumulation to Prevent Autoimmunity
Source: PLoS Pathog. 2010 Sep 16;6(9):e1001111. doi: 10.1371/journal.ppat.1001111 (PMC2940742; doi:10.1371/journal.ppat.1001111)

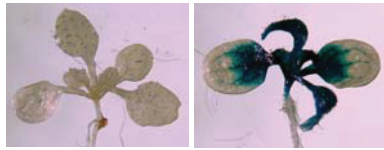

*npr1-1*

*snr5-1 npr1-1*

Supplement: Figure S1 — GUS staining of npr1-1 and snc5-1 npr1-1. Two-week-old seedlings grown on MS media were stained for GUS activity. Both npr1-1 and snc5-1 npr1-1 contain the BGL2 (PR2) Promoter-GUS reporter gene. (0.22 MB PDF) [file ppat.1001111.s001.pdf]

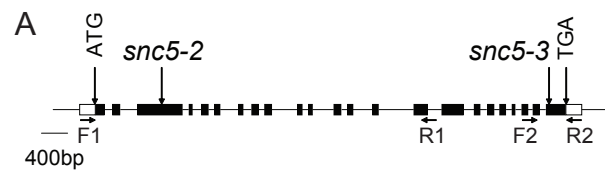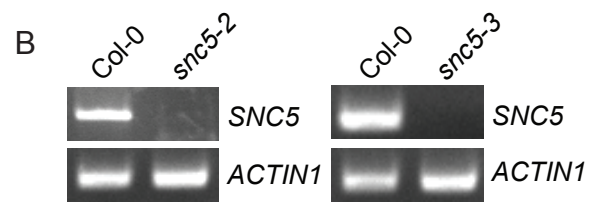

Supplement: Figure S2 — Location of the T-DNA insertions (A) and semi-quantitative RT-PCR analysis of SNC5 expression in the T-DNA knockout mutants snc5-2 and snc5-3 (B). Primers F1 and R1 were used in PCR amplification of snc5-2. Primers F2 and R2 were used in PCR amplification of snc5-3. The locations of the primers are indicated in (A). (0.26 MB PDF) [file ppat.1001111.s002.pdf]

A

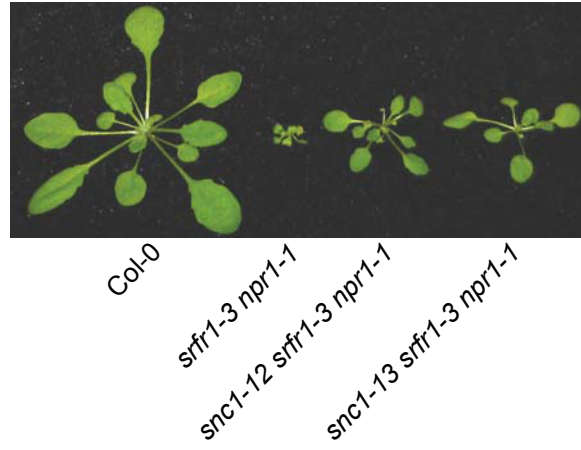

B

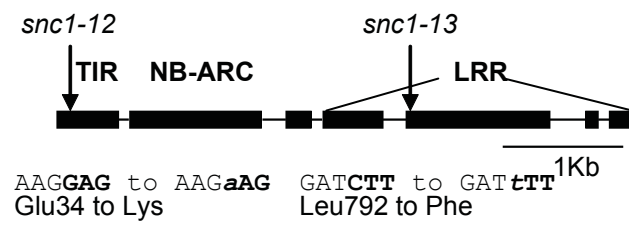

Supplement: Figure S4 — Two suppressor mutants of srfr1-3 npr1-1 carrying mutations in SNC1. (A) Morphology of snc1-12 srfr1-3 npr1-1 and snc1-13 srfr1-3 npr1-1. (B) Molecular lesions in SNC1 identified from snc1-12 and snc1-13. (0.40 MB PDF) [file ppat.1001111.s004.pdf]

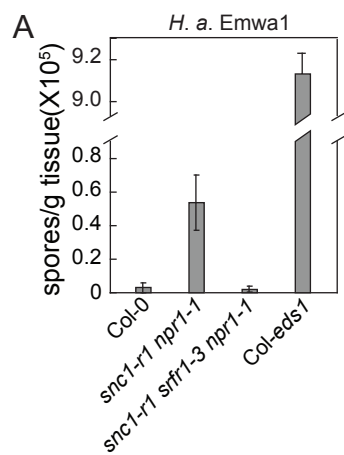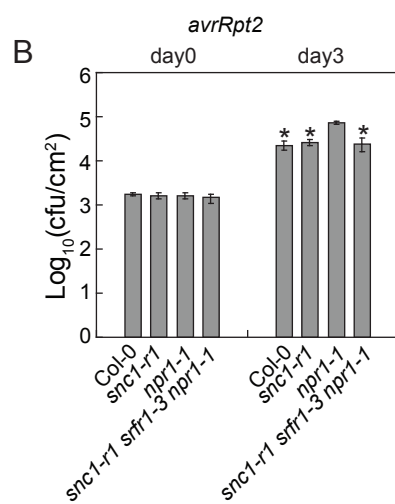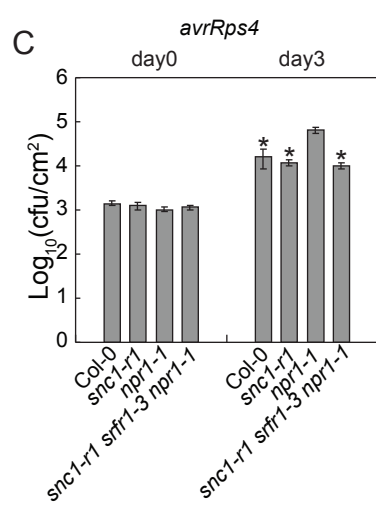

Supplement: Figure S5 — Immunity mediated by RPP4, RPS2 and RPS4 is enhanced in snc1-r1 srfr1-3 npr1-1. (A) Growth of H. a. Emwa1 on WT (Col-0), snc1-r1 npr1-1, snc1-r1 srfr1-3 npr1-1, and eds1-2 (Col). Two-week-old seedlings were sprayed with H. a. Emwa1 at a concentration of 50,000 spores per ml water. Infection was scored 7 days after inoculation by counting the number of spores per gram of tissue. Error bars represent standard deviations from three measurements. (B-C) Growth of P.s.t. DC3000 avrRpt2 (B) and P.s.t. DC3000 avrRps4 (C) on WT (Col-0), snc1-r1, npr1-1 and snc1-r1 srfr1-3 npr1-1. Leaves of five-week old plants were infiltrated with P.s.t. DC3000 carrying avrRpt2 or avrRps4 (OD600 = 0.001). Bacterial growth was determined at Day 0 and Day 3. The values presented are averages of six replicates ± standard deviations (SD). *, P<0.001, significant difference from npr1-1. (0.25 MB PDF) [file ppat.1001111.s005.pdf]

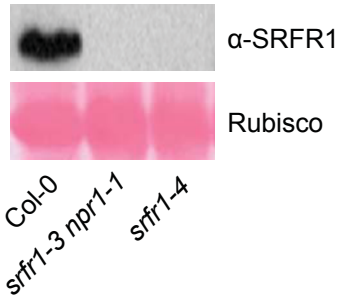

Supplement: Figure S7 — Western blot analysis of the SRFR1 protein in wild type and snc5 mutants using the anti-SRFR1 antibody. (0.17 MB PDF) [file ppat.1001111.s007.pdf]

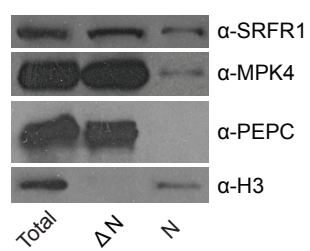

Supplement: Figure S8 — Localization of SRFR1 and MPK4. Immunoblot analysis of SRFR1 and MPK4 in nuclei-depleted (ΔN) and nuclear (N) protein extracts of wild type plants. Equal proportions of nuclei-depleted and nuclear protein extracts were loaded. Anti-PEPC was used as a cytosolic marker, and anti-Histone H3 was used as a nuclear marker. (0.47 MB PDF) [file ppat.1001111.s008.pdf]

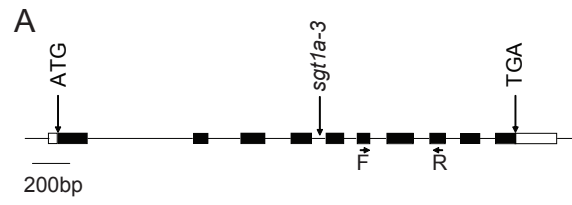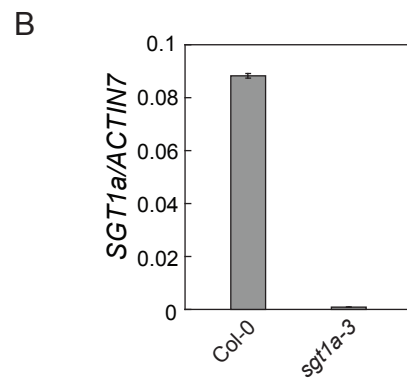

Supplement: Figure S9 — Location of the T-DNA insertion in sgt1a-3 (A) and real-time RT-PCR analysis of SGT1a expression in wild type (Col-0) and sgt1a-3 (B). Primers used for the PCR analysis are indicated in (A). Error bars represent standard deviation from three measurements. (0.23 MB PDF) [file ppat.1001111.s009.pdf]
