# Supplementary material for: SRFR1 Negatively Regulates Plant NB-LRR Resistance Protein Accumulation to Prevent Autoimmunity
Source: PLoS Pathog. 2010 Sep 16;6(9):e1001111. doi: 10.1371/journal.ppat.1001111 (PMC2940742; doi:10.1371/journal.ppat.1001111)
Supplement: Figure S3 — Alignment of SRFR1 and its homologs in rice, human and mouse. AtSRFR1, OsSRFR1, MmSRFR1, and HsSRFR1 represent NP_195462, NP_001058749, NP_663582, and NP_078801 respectively. The sequences were retrieved from NCBI and aligned by the ClustalX2. The aligned data were further analyzed by the BOXSHADE 3.21 (http://www.ch.embnet.org/software/BOX_form.html). (0.84 MB PDF) [file ppat.1001111.s003.pdf]

```
AtSRFR1     1 MATATATSERFELAKHCSSRNWSKAIRVLDSLLAKE-SSILDICNRAFCYNQLELHKHVI
OsSRFR1     1 -----MGSERAELARVCGGRNWSKAIRILDAHLARSPSSIHDLCNRAFCYSQLELHKHVV
MmSRFR1     1 ---------MAPAGCGCGCS------GSLGAAVVRP------------------------
HsSRFR1     1 ---------MAPAGCCCCCCFWGGAVAAAGAARRVL------------------------

AtSRFR1    60 KDCDKALLLEPFAIQAFILKGRALLALGRKQEAVLVLEQGYKSALQQTADVKQLLELEEL
OsSRFR1    56 KDCDRALELDPALLQAYVLKGKALSALGKREEALAVWEQGHEVAVRDTMDLKQLLELEEL
MmSRFR1    22 ----------------------LLLLLG-------ALAC-----ARATEHYSPLSLLKQE
HsSRFR1    28 ----------------------LLLLLG-------VLSAGLRPGALATEHYSPLSLLKQE

AtSRFR1   120 LKDARREIDGILKSHATESPQETPAYHSEKSDEKSDKLDNHESGASSNGNSHESSSELGE
OsSRFR1   116 VSSVK-----ICETIECEDRVVDASPCDTKVVISEDRVVDTSCTATTMAD----------
MmSRFR1    48 LQHRQ--------------QQEAPAGGG--CPQSGDWADQY-PEC---------------
HsSRFR1    59 LQHRQ--------------QQEAPAGGGGCSPQSGDWGDQYSAECG--------------

AtSRFR1   180 QSKIVSFSKVASKASKQSDGNSDLCNGSVYKEKENGKCGSQINGYYESCKPCNGSDLHDN
OsSRFR1   161 -TKTVVCEENIGNSGVISNGAVILAND--NKADNNKECSSPTKDTTGTHHTPKKTTKPDK
MmSRFR1    76 --------------------------------------ESSFLNFHESDCELRGSAPCDS
HsSRFR1    91 --------------------------------------ESSFLNFHDSDCEPKGSSPCDS

AtSRFR1   240 LAESSDRFGELSINGNKISIKSSKMSHKAEARCGISDESRKNKKYTIARISGTHSISVDF
OsSRFR1   218 KSKAK---GRKEINSQIEDVADSISSGETVAVDQTLFASKISK--------SSKSISLDF
MmSRFR1    98 LLSLN----TEKILSQAKSIAEQKRFP-----------------------FATDNDSTNE
HsSRFR1   113 LLSLN----TEKILSQAKSIAEQKRFP-----------------------FATDNDSTNE

AtSRFR1   300 RLSRGIAQVNEGNYTKAISIFDKVLKEEPTYPEALIGRGTAYAFQR-----ELESAIADF
OsSRFR1   267 RLSRGIAQVNEGRYDQAISIFDQILRETPTYPEALIGRGTAYAFQR-----ELDSAISDF
MmSRFR1   131 ELAIAYVLVGSGLYDEAIRHFSTMLQ----------------------------------
HsSRFR1   146 ELAIAYVLIGSGLYDEAIRHFSTMLQEEPDLVSAIYGRGIAYGKKGLHDIKNAELALFEL

AtSRFR1   355 TKAIQSNPAASEAWKRRGQARAALGEYVEAVEDLTKALVFEPNSPDVLHERGIVNFKSKD
OsSRFR1   322 TKAIQSNPSAGEAWKRRGQARAALGEFTEAVEDLTKALEFEPNSPDILHERGIVNFKFKD
MmSRFR1   157 ------------------------------------------------------------
HsSRFR1   206 SRVITLEPDRPEVFEQRAEILSPLGRINEAVNDLTKAIQLQP-SARLYRHRGTLYFISED

AtSRFR1   415 FTAAVKDLSICLKQEKDNKSAYTYLGLAFASLGEYKKAEEAHLKSIQLDSNYLEAWLHLA
OsSRFR1   382 YNAAVEDLSTCVKRDKKNSSAHTYLGLTLSALGEYKRAEDEHLLGIKYDENFLDSWAHLS
MmSRFR1   157 ------------------------------------EAIEAFKEALKQKVDFIDAYKSLG
HsSRFR1   265 YATAHEDFQQSLELNKNQPIAMLYKGLTFFHRGLLKEAIESFKEALKQKVDFIDAYKSLG

AtSRFR1   475 QFYQELADHCKALECIEQVLQVDNRVWKAYHLRGLVFHGLGEHRKAIQELSIGLSIEN-T
OsSRFR1   442 QLYLDIGCPEKMLNNVEKVLQIDVSFGKAYHLRGILYHGMGRHRSAIKDLSVALKHESSN
MmSRFR1   181 QAYRELGNFDAATESFQKALLLNQNHVQTLQLRGMMLYHHGSLQEALKNFKRCLQLEPYN
HsSRFR1   325 QAYRELGNFEAATESFQKALLLNQNHVQTLQLRGMMLYHHGSLQEALKNFKRCLQLEPYN

AtSRFR1   534 IECLYLRGSCYHAVGEYRDAVKDYDATVDVELDAVEKFVLQCLAF-YQKELALYTASKVS
OsSRFR1   502 IECLYLRASCHHAIGEYKAAIKDYDDVLDLELDSMDKFVLQCLAF-YQKEIALYTASKAN
MmSRFR1   241 EVCQYMKGLSHVAMGQFYEGIKAQTKVMLNDPLPGQKASPEYLRVKYLREYSRYLHAHLD
HsSRFR1   385 EVCQYMKGLSHVAMGQFYEGIKAQTKVMLNDPLPGQKASPEYLKVKYLREYSRYLHAHLD

AtSRFR1   593 SEFLCFDIDGDIDPMFKEYWCKRLHPKNVCEKVYRQPPLRESLKKGKLKKQDLAITKQKA
OsSRFR1   561 LEFSQFNIDDDVDPLFKEYWCKRLHPKNVAEKVYRQPPLRISLRSGRLNKQDFKFTKHQT
MmSRFR1   301 TPLTEYNIDSDLPGSFKDHWAKNLP---FLIDGYEEQPGLQPHIRDVLHQNFEGYKPEVQ
HsSRFR1   445 TPLTEYNIDVDLPGSFKDHWAKNLP---FLIEDYEEQPGLQPHIKDVLHQNFESYKPEVQ

AtSRFR1   653 NILRFADLIGKRIQYDCPGFLPNKRQHRMAGLAVIEIAQKVSKAWRIEWRNSTKGTTKNG
OsSRFR1   621 TLILAADSIGKKIQYNCRGFLPNQRQYRMAGLAAIEIAQKVSKAWRFLRN-----PKNNA
MmSRFR1   358 ELICVADRLGSLMQYETPGFLPNKRIHRAMGLAALEVMQAVHRTW-------------TN
HsSRFR1   502 ELICVADRLGSLMQYETPGFLPNKRIHRAMGLAALEVMQAVQRTW-------------TN
```

```
AtSRFR1  713 KKNRRRERTNILSQNRGGAGCSSSSFSETSTGYASLEDRSSGRSILSWQDVYSPAVRWRQ
OsSRFR1  676 KLVRRRDKLN-ACQNRGGY-CSTSTLSGSPT-SSPNEDRISSGISLSWHDVYNIAVKWRQ
MmSRFR1  405 SKVRMNGKTR----------------------------------LLQWRDMFDIAVKWRR
HsSRFR1  549 SKVRMNGKTR----------------------------------LMQWRDMFDIAVKWRR

AtSRFR1  773 ISEPCDPVVWVNKLSEEFNS-GFGSHTPMVLGQAKVVRYFPNYERTLTLAKSIIKDKLSV
OsSRFR1  733 ISEPCDPVVWINKLSEEFNS-GFGSHTPMLLGQAKIIRYYPYYQSVLEAAKNIMLDLKYV
MmSRFR1  431 IADPDQPVLWLDQMPAPSLSRGFNNHINLIRGQVINMRYLEYFEKILHFIKDRILVYHGA
HsSRFR1  575 IADPDQPVLWLDQMPARSLSRGFNNHINLIRGQVINMRYLEYFEKILHFIKDRILVYHGA

AtSRFR1  832 RSKKDKVIDLSKDEKIEKIMRAETCDELHNIVGED-FWVATWCDSTGSEGKRLEGTRITC
OsSRFR1  792 NNAEDRAIFLTDIEKLKKIEVASSCSELYHIVGET-YWVSTRCDSIAFQGRRLEGTRIT-
MmSRFR1  491 NNPKGLLEVREALEKVHKVEDLLPIMKQFNTKTKDGFTVNTKVPSLKDQGKEYDGFTITI
HsSRFR1  635 NNPKGLLEVREALEKVHKVEDLLPIMKQFNTKTKDGFTVNTKVPSLKDQGKEYDGFTITI

AtSRFR1  891 IQKPGRLGYDFSIRTPCTPARWSDFDEEMTSAWEALCTAYCGENYGSTELDALETVRDAI
OsSRFR1  850 TQNMGKTGFDFAIRTPCTPSRWEEYDEEMSAAWEAICEAYCSDTNPTRDPDMLDAVKAAI
MmSRFR1  551 TGDKVGN-ILFSVETQTTEERTQLYHAEIDALYKDLT-AKGKVLTLSAEFGEADAVCNLI
HsSRFR1  695 TGDKVGN-ILFSVETQTTEERTQLYHAEIDALYKDLT-AKGKVLILSSEFGEADAVCNLI

AtSRFR1  951 LRMTYYWYNFMPLARGTAVTGFVVLLGLLLAANMEFTETIPKG-----------------
OsSRFR1  910 LRMTYYWYNFMPLSRGSAVVGYVVLLGLFLAANMDVTASIPHG-----------------
MmSRFR1  609 LSLVYYFYNLMPLSRGSSVIAYSVIVGALMASGKEVAGKIPKGKVCWAGGGTGMAAPRPG
HsSRFR1  753 LSLVYYFYNLMPLSRGSSVIAYSVIVGALMASGKEVAGKIPKG-----------------

AtSRFR1  994 --------------------------LQIDWEAILNVEPGSFVDSVKSWLYPSLKINTSW
OsSRFR1  953 --------------------------VQVDWEAILSQDPDTFVDKIKPWLYPSIKTSRNL
MmSRFR1  669 LLAVDRMRARPHTISFLLSRPAPPLFQLVDFEAMTAPGSEAFSKIAKSWMN-LKSISPSY
HsSRFR1  796 --------------------------KLVDFEAMTAPGSEAFSKVAKSWMN-LKSISPSY

AtSRFR1 1028 RDHTEISSAFSTTGAVVAALSTYND-------
OsSRFR1  987 KDYADVSVAFSTTGSVVAALTCVDT-------
MmSRFR1  728 KTLPSVSETFPTLRSMIEVLNTDSTPRCLKKL
HsSRFR1  829 KTLPSVSETFPTLRSMIEVLNTDSSPRCLKKL
```
