# Supplementary material for: SRFR1 Negatively Regulates Plant NB-LRR Resistance Protein Accumulation to Prevent Autoimmunity
Source: PLoS Pathog. 2010 Sep 16;6(9):e1001111. doi: 10.1371/journal.ppat.1001111 (PMC2940742; doi:10.1371/journal.ppat.1001111)
Supplement: Figure S6 — Yeast two-hybrid analysis of self-association of the TPR domain of SRFR1. (0.28 MB PDF) [file ppat.1001111.s006.pdf]

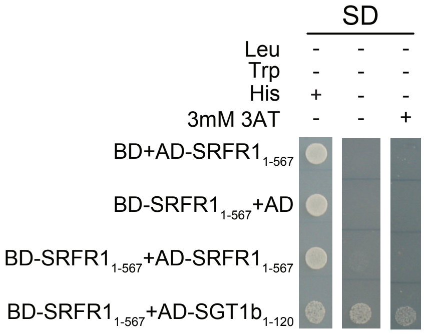
SD
Leu - - -
Trp - - -
His + - -
3mM 3AT - - +
BD+AD-SRFR1 1-567
BD-SRFR1 1-567+AD
BD-SRFR1 1-567+AD-SRFR1 1-567
BD-SRFR1 1-567+AD-SGT1b 1-120
